# Layer wise Scaled Gaussian Priors for Markov Chain Monte Carlo Sampled deep Bayesian neural networks

**DOI:** 10.3389/frai.2025.1444891

**Published:** 2025-04-25

**Authors:** Devesh Jawla, John Kelleher

**Affiliations:** ^1^School of Computer Science, Technological University Dublin, Dublin, Ireland; ^2^ADAPT Research Centre, School of Computer Science and Statistics, Trinity College Dublin, Dublin, Ireland

**Keywords:** Bayesian neural network (BNN), Markov Chain Monte Carlo (MCMC), deep learning artificial intelligence, neural network, Bayesian inference (BI)

## Abstract

Previous work has demonstrated that initialization is very important for both fitting a neural network by gradient descent methods, as well as for Variational inference of Bayesian neural networks. In this work we investigate how *Layer wise Scaled Gaussian Priors* perform with Markov Chain Monte Carlo trained Bayesian neural networks. From our experiments on 8 classifications datasets of various complexity, the results indicate that using *Layer wise Scaled Gaussian Priors* makes the sampling process more efficient as compared to using an Isotropic Gaussian Prior, an Isotropic Cauchy Prior, or an Isotropic Laplace Prior. We also show that the cold posterior effect does not arise when using a either an Isotropic Gaussian or a layer wise Scaled Prior for small feed forward Bayesian neural networks. Since Bayesian neural networks are becoming popular due to their advantages such as uncertainty estimation, and prevention of over-fitting, this work seeks to provide improvements in the efficiency of Bayesian neural networks learned using Markov Chain Monte Carlo methods.

## 1 Introduction

Deep learning has seen a lot of progress in the recent years but lacks the uncertainty quantification required for decision making, especially in safety critical scenarios (Wilson, 2020; Papamarkou et al., 2024). However, Deep Bayesian models are able to provide us with both accurate solutions and uncertainty estimates. Deep Bayesian Learning can be done using a number of Bayesian inference techniques such as Markov Chain Monte Carlo (MCMC), Variational inference (Hinton and van Camp, 1993; Blundell et al., 2015), and Laplace Approximation (Daxberger et al., 2021) methods. MCMC methods are the most accurate and therefore are most useful in safety critical scenarios where accurate solutions and calibrated uncertainty information is sought. There are several MCMC algorithms available, such as No U-Turn Sampling (NUTS) (Hoffman and Gelman, 2011), Hamiltonian Monte Carlo (HMC) (Neal et al., 2011), Metropolis-Hastings Algorithm (Metropolis et al., 1953; Hastings, 1970) and the Particle Gibbs Algorithm (Andrieu et al., 2010).

One of the reasons why deep learning has been successful is the use of He et al. (2015) and/or Glorot and Bengio (2010) initialization.^1^ The majority of neural network training is done using stochastic gradient descent to optimize weights and biases and we call such neural networks as **point neural networks** because after the training is completed with obtain a single neural network as compared to an ensemble of neural networks in a Bayesian Neural network. However, this process of calculating gradients may suffer from instability during the training process due to the problem of vanishing and exploding gradients (Kelleher, 2019). These phenomena have been extensively studied for point neural networks, whose neural network parameters are point estimates, however, relatively little work has been done on studying these phenomena in the context of MCMC methods for Bayesian neural networks whose neural network parameters are distributions. In the literature on Bayesian neural networks we do find the use of *He and/or Glorot priors* (see for example Rossi et al., 2019; Wenzel et al., 2020; Goulet et al., 2020; Noci et al., 2021a), none of these works have studied the effect of these priors on the efficiency of Markov Chain Monte Carlo Sampling. Furthermore, the use of Isotropic Priors still persists in the literature (see Immer et al., 2020; Vladimirova et al., 2018), which we attribute to the lack of systematic empirical analysis of the effects of Isotropic and Layer wise Scaled priors on learning efficiency.

Another interesting phenomenon discussed in the literature on Bayesian neural networks is the Cold posterior effect (CPE) (Aitchison, 2020; Adlam et al., 2020; Noci et al., 2021b) where scaling the posterior of a network results in an improvement in the classification performance of several vision tasks. In the context of safety critical applications it is important that the probabilities generated by the system are well calibrated, however due to CPE the results obtained have an underconfident aleatoric uncertainty representation (Kapoor et al., 2022). The effect of inference method for a Bayesian neural network on the CPE has been examined by Wenzel et al. (2020) who argue that the cold posterior effect is observed regardless of the inference technique used. However, in a subsequent study Izmailov et al. (2021) found that no cold posterior effect is observed when reproducing the Wenzel et al. (2020) study with an implementation of their own HMC, and they argue that CPE is largely an artifact of data augmentation.

Prior mis-specification is another cause studied in the CPE literature (Izmailov et al., 2021; Wenzel et al., 2020). Fortuin et al. (2021) propose using heavy tailed priors instead of the Gaussian priors to avoid the CPE. However Gaussian priors are convenient to conceptualize (because if we consider our likelihood to be normally distributed then the Gaussian Prior is a conjugate prior) and therefore they are routinely used in the literature. In research on Bayesian neural networks it is common practice (see e.g., Immer et al., 2020; Vladimirova et al., 2018) to specify the weights of the neural network as an isotropic Gaussian prior, which is a multivariate Normal distribution of mean 0 and variance 1.

In this work we compare the efficiency of using Layer wise Scaled Gaussian Priors with Isotropic Gaussian Prior, Laplace Prior and the Cauchy Prior for the weights of a Dense layer Bayesian Neural Network. We use the MCMC for collecting an ensemble of trained neural networks because MCMC is a very accurate sampling algorithm which samples directly from the true posterior (for more therotical details about Bayesian neural networks and Bayesian Inference techniques see Appendix A.1). We check by comparing classification performance, efficiency in time, and MCMC convergence statistics the suitability of the Priors on neural network parameters. We provide experimental results, and study the dynamics of bayesian neural networks of placing different priors on the parameters. Lastly we study the CPE when using Isotropic and Layer wise Scaled Gaussian Priors, and we investigate class imbalance as the probable cause of the cold posterior effect.

## 2 Experiment details

### 2.1 Datasets

**Figure 1:**
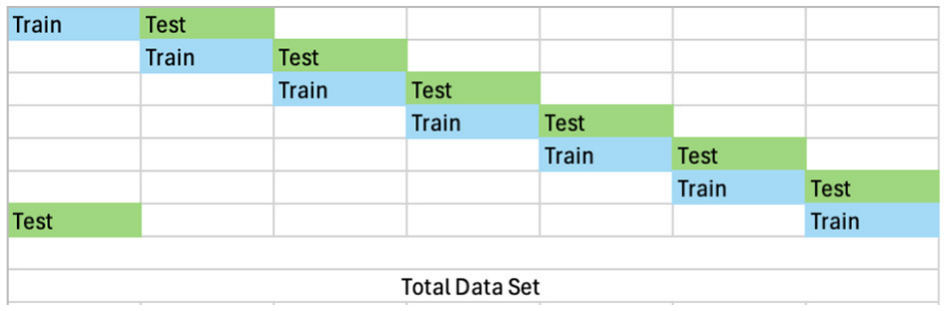
Train test splits.

For the experiments conducted in this study we choose the following eight data sets: Adult (Becker and Kohavi, 1996), Fake Banknote detection (Lohweg, 2013), Credit Fraud Detection (Dal Pozzolo et al., 2017), Stroke detection (Kansadub et al., 2015), Credit default detection (Yeh, 2016), Coalmine Coalmine bumps detection (Sikora and Wrobel, 2010), Iris classification (Fisher, 1988), and Yeast classification (Nakai, 1996). We have selected these tabular data sets considering the resource-intensive nature of the Bayesian Inference methods. For all the datasets we split the data into equal and independent train:test splits, see Table 1 for more details, where for a ten fold train:test split each train split is unique and each test is unique. Figure 1 shows the prescription used to achieve this. We do this to keep the computation costs to a minimum and to obtain independent and uncorrelated estimates.

**Table 1:** K-Fold splits, query sizes, train sizes and class imbalance of the eight real data sets.

**Data set**	**% Minority class**	**Folds**	**Train:test per fold**	**Total samples**
Adult	0.22	10	1,000:1,000	48,720
Banknote	0.44	10	137:137	1,377
Credit fraud	0.17	10	1,000:1,000	284,767
Credit default	0.22	10	1,000:1,000	29,920
Coalmine	0.06	10	258:258	2,584
Stroke	0.05	10	490:490	4,916
Iris	0.3	5	30:30	150
Yeast	0.003	10	149:149	1,496

### 2.2 Data pre-processing

We perform minimal data pre-processing,^2^ and only remove redundant features, rows with missing values and NaN values. For the continuous input features, we either perform MinMaxScaling for features with known upper and lower boundaries from domain knowledge, or we perform a MaxAbsScaling (division by the maximum value of the feature) for sparse features, and we perform Normalization (transform the feature values to have zero mean and unit variance) for continuous features which belong to neither of the two previous categories of continuous features and are normally distributed. An example of a redundant feature is when it does not offer any information, for example if all samples have the same value. Lastly, we perform one hot encoding of the categorical features.

### 2.3 Class weighted loss or likelihood

To account for the class imbalance we use a weighting scheme which we call as **the class weighted likelihood** in the case of Bayesian Inference, and **the class weighted loss** in the case of the Stochastic gradient descent training. It can be implemented by multiplying each term in the likelihood (see Equation 4 on page 13) for the corresponding sample of the *i*^*th*^ class in the training data set by the following coefficient:


(1) $$\begin{array}{l} \frac{\text{Total number of Training samples}}{\text{Samples of the class of }i^{th}\text{ sample}\times \text{Classes}} \end{array}$$

This has the effect that the class with the highest proportion in the training set is weighted by a factor which is smallest, thereby reducing the contribution of this sample in the loss function. The cumulative effect of this weighted likelihood is that samples of each class contribute equally to the loss function.

### 2.4 Models

We use Flux.jl (Innes, 2018) to design the neural networks and we use Turing.jl (Ge et al., 2018) to define the Bayesian Models with the appropriate priors and the likelihood. We define the Prior on all the model weights as a Multivariate Normal Distribution with a mean and variance as hyper-parameter defined by the respective experiments (for example Layer wise scaled, Isotropic, Identity etc). The prior on the *n* neural network parameters (weights and biases) is $\theta_{n}=N(\mu ,\sigma^{2})$ where **μ** = [0_1_…0_*n*_] and $\sigma^{2}=[\sigma_{1}^{2}\ldots \sigma_{n}^{2}]$. The @model macro of the Turing package adds the log joint probability internally for each sample, and the posterior (the product of the likelihood and the Prior) is internally defined, in accordance with the Bayes Theorem. Therefore, all the user needs to do is choose a Markov Chain Monte Carlo sampler to sample from the posterior.

We choose the No U Turn Sampler (NUTS) (Hoffman and Gelman, 2011) algorithm which is a state of the art. We run 8 chains and we sample for 200 Monte Carlo steps after the burn-in period of 1000 MC steps. We then use thinning, keeping only every fourth sample in each chain. This gives us an ensemble of 400 independent neural networks (estimators).

We use a neural network which contains two hidden layers with 12 neurons in each layer, where all the neurons in the hidden layer have a ReLU activation function. Finally we omit using the bias in the output layer neurons. Although relatively small, this neural network is in line with the current literature (Immer et al., 2020; Kessler et al., 2023; Izmailov et al., 2021; Gudur et al., 2019) and provides classification accuracy comparable to the state of the art.

### 2.5 Evaluation metrics

To benchmark the classification performance we use Balanced Accuracy for all the data sets,and MacroF1Score. And to benchmark the efficiency of the models we measure the time required to sample the total number of Monte Carlo (MC) samples.

To evaluate for convergence of the chains, we look at the following metrics:

1. **Gelmann diagnostic** (Vehtari et al., 2019)—Convergence statistic which tells us how well the chains have mixed, it compares the between- and within-chain estimates for weights and biases.
2. **OOB (out of bounds) $\hat{R}$**—which tells us how many parameters have $\hat{R}$ values which are greater than 1.01 and less than 0.99. This number should be zero for a well converged chain.
3. **Average acceptance rate**—The percentage of proposals which are accepted on average during a sampling step, this should be high or comparable to what is expected from the chosen Sampler.
4. Total numerical error—The number of numerical errors encountered during the sampling process
5. Avg ESS (effective sample size) (Geyer, 1992)—it is an indication of the number of independent samples contained in a chain
6. Elapsed—It is the amount of time taken to sample a fixed length of the Markov Chain.

## 3 Experiments

### 3.1 Layer wise Scaled Gaussian Priors vs. Isotropic Priors

In this experiment we examine if using a *Layer wise Scaled Gaussian Prior* (Gaussian Prior with mean 0 and variance specified per layer using a Glorot or He Initialization) is more efficient than *Isotropic Priors* (Gaussian Prior, Laplace Prior, and a Cauchy Prior whose locations are 0 and scales 1) for Bayesian neural networks trained using MCMC. We chose these Isotropic Priors as baseline comparisons because together they cover a representative range of tail heaviness for reasonable unimodal priors (see Figure 1 of the Appendix for a visual comparison of the distributions). The specific form of Layer wise scaled prior used is dependent on the activation function used in the layer, we use a *He Prior* for ReLU activated layers or a *Glorot Prior* for Sigmoid/Tanh activated layers. For example, with a *Glorot Prior* each weight in the layer is a Normal distribution with mean 0 and a variance calculated using Glorot-Initialization (Glorot and Bengio, 2010).

Each of the components of an Isotropic Gaussian Prior placed on the neural network parameters are defined as $N\left(\mu =0,\sigma^{2}=1\right)$. Similarly, each of the components of an Isotropic Laplace Prior and the Isotropic Cauchy Prior placed on the neural network parameters are defined as L(μ=0,b=1) and $C\left(x_{0}=0,\gamma =1\right)$ respectively. The components of the layer-wise scaled Gaussian Prior are defined layer by layer for the *n* layers as $\text{N}\left(\mu =0,\sigma_{Glorot}^{2}\right)$ where $\sigma_{Glorot}^{2}=\left[\sigma_{1}^{2}\ldots \sigma_{n}^{2}\right]$ are the variances of the parameters in the respective layers as follows


$$\sigma_{Glorot}^{2}=16\frac{2}{n_{in}+n_{out}}$$

where *n*_*in*_ is the number of input connections and *n*_*out*_ is the number of output connections for a neuron in a given layer.

From the results of our experiments given in Table 2, we see that the Layer wise Scaled Gaussian Prior is more efficient (with statistically significant results highlighted in bold font) than all of the Isotropic Priors, for all eight datasets and this gain in efficiency does not come at a cost of classification performance and we see comparable or better classification performance (with results which are better but are not statistically significant, highlighted in Italic font). The ± signify one standard deviation across five measurements for each dataset respectively.

**Table 2:** Comparison of efficiency and performance for different priors.

**Dataset**	**Prior**	**BalAcc**	**MacroF1**	**Time**
Adult	Iso. Cauchy	0.71 ± 0.05	0.54 ± 0.02	106.66 ± 0.94
	Iso. Laplace	0.74 ± 0.03	0.55 ± 0.01	31.99 ± 4.31
	Iso. Gaussian	0.74 ± 0.03	0.56 ± 0.01	17.37 ± 0.58
	L.S. Gaussian	0.76 ± 0.03	0.51 ± 0.03	**5.79** **±0.2**
Banknote	Iso. Cauchy	0.93 ± 0.04	0.92 ± 0.04	90.14 ± 0.63
	Iso. Laplace	0.94 ± 0.04	0.94 ± 0.04	72.55 ± 7.8
	Iso. Gaussian	0.95 ± 0.03	0.94 ± 0.03	32.81 ± 5.24
	L.S. Gaussian	0.94 ± 0.05	0.94 ± 0.05	**9.43** **±0.26**
Coalmine	Iso. Cauchy	0.69 ± 0.09	0.16 ± 0.03	164.32 ± 1.86
	Iso. Laplace	0.71 ± 0.07	0.17 ± 0.03	67.73 ± 18.28
	Iso. Gaussian	0.74 ± 0.02	0.17 ± 0.02	31.56 ± 7.81
	L.S. Gaussian	0.74 ± 0.03	0.15 ± 0.01	**7.98** **±0.23**
Credit Default	Iso. Cauchy	0.65 ± 0.05	0.43 ± 0.02	184.03 ± 1.16
	Iso. Laplace	0.64 ± 0.07	0.43 ± 0.03	116.81 ± 23.34
	Iso. Gaussian	0.64 ± 0.04	0.43 ± 0.02	56.14 ± 1.38
	L.S. Gaussian	0.71 ± 0.08	0.45 ± 0.05	**12.11** **±4.71**
Credit Fraud	Iso. Cauchy	0.94 ± 0.03	0.07 ± 0.07	160.88 ± 3.1
	Iso. Laplace	0.94 ± 0.05	0.17 ± 0.3	142.79 ± 8.49
	Iso. Gaussian	0.94 ± 0.04	0.15 ± 0.24	107.08 ± 31.16
	L.S. Gaussian	**0.99** **±0.01**	0.23 ± 0.11	**21.32** **±5.33**
Iris	Iso. Cauchy	0.87 ± 0.12	0.85 ± 0.16	89.84 ± 1.07
	Iso. Laplace	**0.89** **±0.13**	**0.88** **±0.17**	21.16 ± 3.7
	Iso. Gaussian	0.86 ± 0.13	0.85 ± 0.17	12.32 ± 2.75
	L.S. Gaussian	0.65 ± 0.19	0.6 ± 0.22	**3.91** **±0.68**
(1000 MC steps)	L.S. Gaussian	0.75 ± 0.08	0.7 ± 0.13	41.33 ± 13.82
Stroke	Iso. Cauchy	**0.75** **±0.06**	0.17 ± 0.03	109.43 ± 6.52
	Iso. Laplace	0.74 ± 0.06	0.17 ± 0.03	32.89 ± 4.92
	Iso. Gaussian	0.73 ± 0.07	0.17 ± 0.02	19.68 ± 5.55
	L.S. Gaussian	0.58 ± 0.08	0.15 ± 0.02	**7.76** **±4.93**
Yeast	Iso. Cauchy	0.44 ± 0.07	**2.09** **±1.43**	534.72 ± 8.76
	Iso. Laplace	**0.45** **±0.07**	1.87 ± 1.21	845.2 ± 743.58
	Iso. Gaussian	**0.45** **±0.09**	1.9 ± 1.22	492.84 ± 50.64
	L.S. Gaussian	0.34 ± 0.11	1.39 ± 1.04	**76.56** **±3.21**
(1000 MC Steps)	L.S. Gaussian	0.4 ± 0.1	1.56 ± 1.07	752.21 ± 49.58

Values in Bold indicate the top one, two or three competing results obtained for a given dataset.

From the analysis of the convergence statistics (see Section 2.5 for more information) collected for a total of 100 Monte Carlo Steps (Table 3) of the NUTS algorithm, we observe the following results:

1. The Out of Bounds $\hat{R}$ is the lowest for Layer wise scaled Gaussian Prior signaling a better convergence (We note here however that this number should be zero ideally and we can achieve this if we let the Markov Chain run longer, for example for 1,000–5,000 Monte Carlo Steps)
2. There are no numerical errors when using an Isotropic Gaussian Prior or an Layer wise scaled Gaussian Prior, however we do get numerical errors when using heavy tailed priors such as Isotropic Laplace and Isotropic Cauchy Prior
3. The ESS is highest (with statistically significant results in **Bold**) for Layer wise scaled Gaussian Prior and signifies that the Prior is more suitable than the Isotropic Priors
4. PSRF, Gelmann Diagnostic should be ideally 1.00 ± 0.01, and we see that Layer wise scaled Gaussian Prior have values closest to 1. As a control experiment, we let the Markov chain run for 1000 Monte Carlo steps for the Iris and Yeast Datasets in the case of Layer wise Scaled Gaussian Prior, and as expected we observe the OOB $\hat{R}$ to drop significantly, and the PSRF Gelman diganostic to have become 1.01 and 1.02 respectively. These two metrics signify that the chains have converged, as they should, eventually.

**Table 3:** Comparison of efficiency and performance for different priors.

**Dataset**	**Experiment**	**OOB $\hat{R}$**	**Numerical Errors**	**ESS**	**PSRF**
Adult	Iso. Cauchy	189.8 ± 14.89	0.4 ± 0.22	0.17 ± 0.01	7.16 ± 2.02
	Iso. Laplace	140.8 ± 8.04	0.0 ± 0.0	1.3 ± 0.09	1.22 ± 0.04
	Iso. Gaussian	109.3 ± 5.04	0.0 ± 0.0	2.87 ± 0.46	1.1 ± 0.02
	L.S. Gaussian	**98.2** **±3.33**	0.0 ± 0.0	**14.17** **±1.65**	1.1 ± 0.02
Banknote	Iso. Cauchy	210.9 ± 4.35	0.8 ± 0.91	0.09 ± 0.01	8.2 ± 1.81
	Iso. Laplace	143.1 ± 7.14	0.1 ± 0.22	0.54 ± 0.05	1.23 ± 0.04
	Iso. Gaussian	121.7 ± 9.66	0.0 ± 0.0	1.03 ± 0.06	1.15 ± 0.05
	L.S. Gaussian	**102.7** **±8.55**	0.0 ± 0.0	**6.17** **±0.5**	1.13 ± 0.04
Coalmine	Iso. Cauchy	265.83 ± 1.04	1.0 ± 1.73	0.08 ± 0.01	8.74 ± 1.41
	Iso. Laplace	185.5 ± 17.35	0.17 ± 0.29	0.6 ± 0.17	1.23 ± 0.05
	Iso. Gaussian	161.67 ± 9.36	0.0 ± 0.0	1.19 ± 0.16	1.18 ± 0.03
	L.S. Gaussian	**132.83** **±8.95**	0.0 ± 0.0	**9.09** **±1.37**	**1.1** **±0.03**
CreditDefault	Iso. Cauchy	401.9 ± 9.76	0.5 ± 1.12	0.04 ± 0.0	10.43 ± 2.17
	Iso. Laplace	278.8 ± 17.56	0.0 ± 0.0	0.32 ± 0.06	1.28 ± 0.1
	Iso. Gaussian	216.6 ± 21.93	0.0 ± 0.0	0.75 ± 0.14	1.19 ± 0.11
	L.S. Gaussian	*210.7 ± 19.36*	0.0 ± 0.0	**4.6** **±1.08**	*1.16 ± 0.04*
CreditFraud	Iso. Cauchy	465.7 ± 12.69	4.0 ± 7.62	0.04 ± 0.01	9.99 ± 1.85
	Iso. Laplace	346.4 ± 30.52	0.1 ± 0.22	0.21 ± 0.07	1.32 ± 0.16
	Iso. Gaussian	258.4 ± 11.88	0.0 ± 0.0	0.42 ± 0.07	1.16 ± 0.04
	L.S. Gaussian	**232.8** **±16.14**	0.0 ± 0.0	**4.02** **±0.98**	*1.12 ± 0.02*
Iris	Iso. Cauchy	201.2 ± 6.69	2.2 ± 2.84	0.22 ± 0.02	9.21 ± 4.19
	Iso. Laplace	149.2 ± 6.71	0.1 ± 0.22	2.27 ± 0.52	1.22 ± 0.04
	Iso. Gaussian	**110.2** **±9.84**	0.0 ± 0.0	5.02 ± 0.88	1.1 ± 0.01
	L.S. Gaussian	**110.8** **±8.53**	0.0 ± 0.0	**15.85** **±3.84**	1.1 ± 0.01
(1000 steps)	L.S. Gaussian	**5.9** **±1.85**	0.0 ± 0.0	**15.73** **±4.75**	**1.01** **±0.0**
Stroke	Iso. Cauchy	190.2 ± 7.06	0.3 ± 0.45	0.17 ± 0.02	6.98 ± 2.89
	Iso. Laplace	140.2 ± 7.8	0.0 ± 0.0	1.4 ± 0.3	1.21 ± 0.04
	Iso. Gaussian	102.4 ± 6.54	0.0 ± 0.0	3.12 ± 0.96	1.11 ± 0.05
	L.S. Gaussian	*99.9 ± 7.89*	0.0 ± 0.0	**12.88** **±3.25**	*1.09 ± 0.01*
Yeast	Iso. Cauchy	337.4 ± 6.02	0.4 ± 0.42	0.02 ± 0.0	10.53 ± 3.09
	Iso. Laplace	287.7 ± 8.07	0.0 ± 0.0	0.03 ± 0.02	2.19 ± 0.34
	Iso. Gaussian	235.1 ± 11.82	0.0 ± 0.0	0.05 ± 0.0	1.58 ± 0.36
	L.S. Gaussian	**207.8** **±14.11**	0.0 ± 0.0	**0.44** **±0.06**	**1.31** **±0.09**
(1000 Steps)	L.S. Gaussian	**20.6** **±4.56**	0.0 ± 0.0	**0.36** **±0.1**	**1.02** **±0.0**

Values in Bold indicate the top one, two or three competing results obtained for a given dataset.

### 3.2 Effect of varying the variance of the Isotropic Gaussian prior

The results of our first experiment indicate that using a Layer wise Scaled Gaussian (LSG) Prior results in a more efficient MCMC training process than the Isotropic Priors. Moreover since we obtain poor convergence statistics for the Isotropic Laplace and the Isotropic Cauchy distribution we limit our study in this experiment to the two cases of the Gaussian Priors. In this experiment we check how scaling the variance of the Isotropic Gaussian Prior by a constant affects the efficiency, numerical errors, acceptance rate, and the Balanced Accuracy. We experiment with the Iris and the Stroke datasets, with the following set of Isotropic Variances: 0.01, 0.2, 1.0, 3.0, 5.0 and LSG.

From the results given in Tables 4, 5, we see that using a larger variance leads to an inefficient convergence process and results in an increased sampling time. For all of the variances we test with a Isotropic Gaussian prior, the acceptance rate is low, indicating inefficient sampling. When using a very small variance such as 0.01, we end up with parameters which are too similar, this affects the learning ability of the model and the classification balanced accuracy drops significantly. Layer wise Scaled Gaussian Prior provides us with a good variance of the neural network weights as a default starting point, without us having to manually find the suitable values of the variances for each of the neural network parameters.

**Table 4:** Experiment on the effect of scaling the variance of the prior : iris dataset.

**Variance**	**Acceptance rate**	**Numerical error**	**Balanced accuracy**	**Elapsed (sec)**
0.01	0.72 ± 0.03	0.00	0.35 ± 0.02	65 ± 66
0.2	0.74 ± 0.03	0.00	0.39 ± 0.14	9 ± 7
1.0	0.80 ± 0.03	0.9 ± 0.9	**0.96** **±0.02**	38 ± 2
3.0	0.62 ± 0.04	85.8 ± 39.0	0.95 ± 0.02	117 ± 21
5.0	0.57 ± 0.05	97.8 ± 22.1	0.95 ± 0.02	177 ± 29
LSG	**0.87** **±0.03**	**0.00**	**0.95** **±0.01**	**20** **±5**

Values in Bold indicate the top one, two or three competing results obtained for a given dataset.

**Table 5:** Experiment on the effect of scaling the variance of the prior : stroke dataset.

**Variance**	**Acceptance rate**	**Numerical error**	**Balanced accuracy**	**Elapsed (sec)**
0.01	0.70 ± 0.02	0.00	0.59 ± 0.51	15 ± 2
0.2	0.75 ± 0.01	0.00	0.54 ± 0.39	12 ± 2
1.0	0.80 ± 0.03	0.6 ± 0.7	**0.71** **±0.03**	107 ± 9
3.0	0.72 ± 0.09	9.1 ± 5.7	0.69 ± 0.02	421 ± 8
5.0	0.76 ± 0.07	3.5 ± 2.3	0.70 ± 0.03	426 ± 5
LSG	**0.83** **±0.02**	**0.00**	**0.71** **±0.03**	**60** **±8**

Values in Bold indicate the top one, two or three competing results obtained for a given dataset.

### 3.3 Understanding the internal dynamics of Bayesian networks with different priors

In this experiment we analyze the distributions of the neuron activations and the weights of a Bayesian neural network layer by layer for various sampling lengths (number of Monte Carlo Steps). Observing the violin plots and the box plots of the values of the activations and weights layer by layer helps us understand the variance of the information flow across the network. From the literature we know for point neural networks that gradient descent methods encounter the problem of vanishing and exploding gradients and therefore consistent variance of the activations and weights and biases across the network are important for the training process (Kelleher et al., 2020). Therefore in this experiment we investigate the relationship between the variance of the activations across the layers and the efficiency of the sampling process.

**Figure 2:**
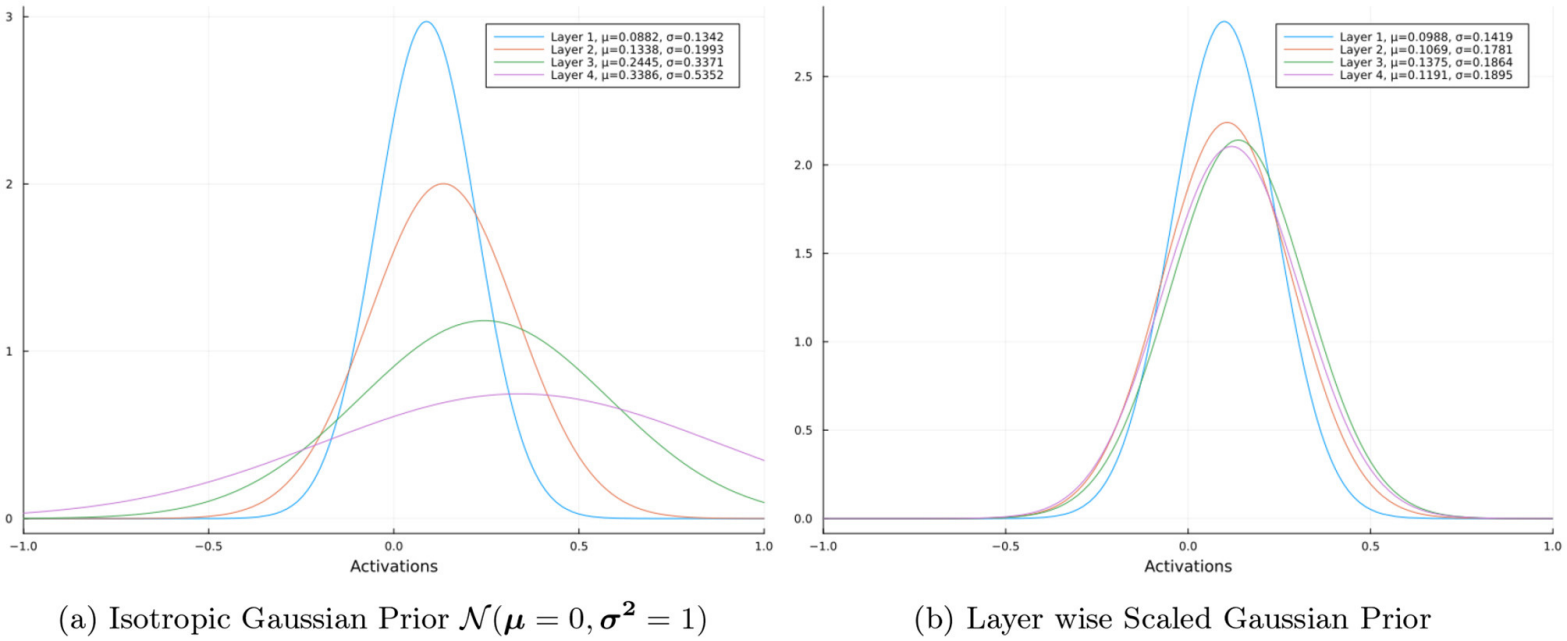
Distribution of layer activations.

Firstly, we look at the distributions of the magnitude of activations of the neurons in each hidden layer for a random input on an untrained neural network. We do this for a 4 hidden layer neural network with ReLU activated layers, where each layer contains 100 neurons. However the for the distributions we only take the activations into consideration without the activation function applied to the logits. Figure 2 shows the distributions of the magnitude of activations of the neurons in each hidden layer of the network for an Isotropic Gaussian prior and a Layer wise Scaled Gaussian Prior respectively. We can clearly observe that the activations of the deeper layers have a larger variance in the case of an Isotropic Gaussian prior. Using Layer wise Scaled Gaussian Priors keeps the variances of the activations across the layers consistent (rather than vanishing or exploding) and this results in an efficient training process.

**Figure 3:**
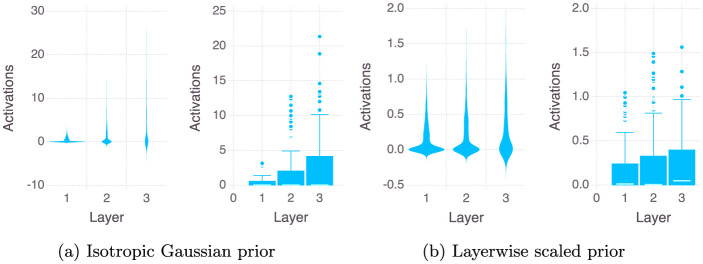
Layer-wise distribution of ReLU activations after 2,000 Monte Carlo steps.

**Figure 4:**
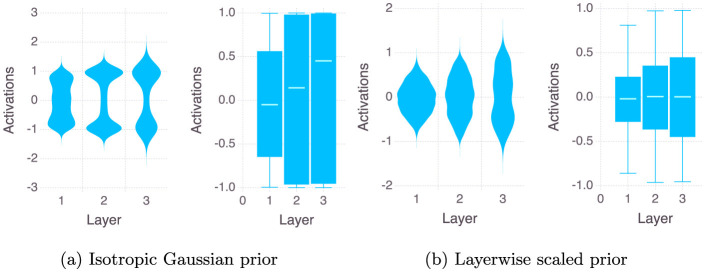
Layer-wise distributions of TanH activations after 2,000 Monte Carlo steps.

Secondly, we compare the case of using a Isotropic Gaussian prior versus a Layer wise Scaled Gaussian Prior for sampling lengths of 10, 200, 1,000, and 2,000 Monte Carlo steps. In the figure we consider here the last sample of the MCMC, i.e. one neural network. We repeat all the experiments on two sizes of the neural network, one being our original two hidden layer neural network with 12 neurons in each layers, the second is a wider neural network with three hidden layers and 24 neurons in each hidden layer. Figures 3, 4 show the violin and the box plots of outputs of the neurons of each of the three layers of the network for the Stroke Dataset.^3^ When using the Isotropic Gaussian prior for a neural networks with ReLU activation function across the hidden layers, from Figure 3 we see that the variance of the activations increases as we go deeper, this effect reduces when we use a layer wise scaled prior. We also note that vertical axes showing the magnitude of the activations are also very different for the two cases, for the Isotropic case being very large almost ten times larger than the Layerwise scaled case.

For a TanH activated neural network, from Figure 4 we see that the activations tend to saturate on the extreme values of −1 and 1, thereby giving us a bimodal type of distribution. We observe that using the Layer wise Scaled Gaussian Prior with TanH activated layers makes the distributions of the activations more Gaussian-like i.e the mass of the distributions concentrates in the center instead of the two extremes at -1 and 1. This is remedied when we use a layer wise scaled prior and we obtain Gaussian-like unimodal distributions for the activation values across all the layers.

### 3.4 Cold posterior effect

In this experiment we continue with the Isotropic and layer-wise scaled Bayesian neural networks but cool the posterior by tempering the likelihood by a factor of 10, and 100 respectively. We are then able to compare in one place all the six different cases, Isotropic Gaussian prior with temperatures 1.0, 0.1, 0.01, and Layer wise Scaled Gaussian Prior with temperature 1.0, 0.1, 0.01 respectively to check if the cold posterior effect is observed.

From the results of our experiments, given in Table 6, when using an Isotropic Gaussian prior we observe no cold posterior effect in any of the eight classification datasets. In fact both the classification performance and efficiency degrade as we cool the posteriors, and our results agree with that of Izmailov et al. (2021). In the case of *Layer wise Scaled Gaussian Priors*, we observe the cold posterior effect only in the case of the Iris dataset and the Yeast dataset. We suspect class imbalance to be the cause for this because for the binary tasks we have used a balanced dataset by undersampling the majority class and we observe no cold posterior effect for either Isotropic or Layer wise Scaled Gaussian Prior, however in the case of the multi-label datasets, the Iris and the Yeast datasets, we do no such curation (undersampling) and it maybe that the scaled posterior fits to the majority classes. In Table 6, we show the results of our experiments using a class weighted likelihood (CWL) without cooling the posterior (i.e., Temperature = 1.0) and we observe that for the two multi-label classification datasets Iris and Yeast, the classification performance improves significantly and performs better than the cooled posteriors of the un-weighted likelihood.

**Table 6:** Experiment on the cold posterior effect for the Isotropic Gaussian prior and Layer wise Scaled Gaussian prior.

**Prior**		**Isotropic**		**Layer-wise Scaled**	
	**Temp**.	**Bal. Acc**.	**Time (sec)**	**Bal. Acc**.	**Time (sec)**
Adult	1.0	**0.75** **±0.02**	116 ± 3	**0.74** **±0.01**	**16** **±1**
	0.1	0.68 ± 0.01	382 ± 34	0.74 ± 0.02	58 ± 3
	0.01	0.70 ± 0.02	382 ± 10	0.68 ± 0.02	335 ± 27
Banknote	1.0	0.98 ± 0.01	75 ± 19	0.98 ± 0.01	**8.7** **±0.2**
	0.1	0.98 ± 0.01	79 ± 19	0.98 ± 0.01	15.1 ± 0.3
	0.01	0.98 ± 0.01	41 ± 20	0.98 ± 0.01	18 ± 4
Credit fraud	1.0	0.97 ± 0.02	1120 ± 13	0.97 ± 0.01	**341** **±277**
	0.1	0.97 ± 0.02	916 ± 108	0.97 ± 0.02	52 ± 14
	0.01	0.96 ± 0.02	308 ± 171	0.96 ± 0.03	81 ± 11
Stroke	1.0	0.71 ± 0.03	85 ± 8	0.67 ± 0.04	**17** **±6**
	0.1	0.66 ± 0.03	285 ± 13	0.71 ± 0.02	44 ± 4
	0.01	0.68 ± 0.01	278 ± 8	0.68 ± 0.03	199 ± 27
Credit default	1.0	0.69 ± 0.04	149 ± 40	**0.74** **±0.05**	**19** **±14**
	0.1	0.66 ± 0.03	345 ± 66	0.64 ± 0.04	82 ± 24
	0.01	0.66 ± 0.02	280 ± 20	0.61 ± 0.03	144 ± 33
Coalmine	1.0	**0.65** **±0.05**	128 ± 23	**0.73** **±0.08**	**19** **±1**
	0.1	0.59 ± 0.01	515 ± 28	0.66 ± 0.01	93 ± 4
	0.01	0.61 ± 0.01	443 ± 64	0.57 ± 0.02	293 ± 13
Iris	1.0	0.8 ± 0.09	14.68 ± 2.93	0.44 ± 0.14	6.62 ± 4.92
	0.1	0.88 ± 0.12	21.97 ± 8.99	0.87 ± 0.12	11.95 ± 4.36
	0.01	0.88 ± 0.12	33.38 ± 23.35	0.88 ± 0.12	30.31 ± 12.6
	CWL	0.87 ± 0.12	**14.05** **±3.09**	0.67 ± 0.2	**6.73** **±4.44**
Yeast	1.0	0.37 ± 0.05	273.05 ± 33.34	0.14 ± 0.03	42.96 ± 1.73
	0.1	0.38 ± 0.05	1,901.43 ± 2965.44	0.39 ± 0.03	560.85 ± 11.1
	0.01	0.39 ± 0.07	326.12 ± 125.37	0.37 ± 0.04	287.15 ± 106.46
	CWL	0.46 ± 0.08	443.29 ± 101.58	0.33 ± 0.1	**71.11** **±14.62**

Values in Bold indicate the top one, two or three competing results obtained for a given dataset.

## 4 Discussion

During the inference of a Bayesian Neural network using NUTS we encounter the problem of infinite gradients and NaNs which firstly affects the adaptation process, often taking a very long time to find the correct step size. Secondly the convergence process can be very inefficient as well, if during the proposal step at each iteration the algorithm has to reject proposals θ′ or the derivatives of the Hamiltonian with respect to it become undefined.

During the Monte Carlo sampling, if the predicted label during the training process is exactly 0 or 1 then it would produce a log(0) term in the log likelihood thereby producing a numerical error (see Equation 4 in the Appendix). This can happen, for example, during the initial iterations of the MCMC algorithm when the neural network has not learned anything, so for a given classification *y*_*i*_ = 0, it is entirely possible that the neural network outputs ŷ_θ, *i*_ = 1, producing a numerical error. Or for example, the *sigmoid* activation of a very small or a very large number (absolute value larger than 6) could output a 0 or 1 respectively for example *sigmoid*(*z* = 10) = 1. We note that this is because the implementation of the sigmoid function in most of the standard libraries clips the output to either 0 or 1 after a certain point and when this zero is inside the log, this quanity becomes undefined and this produces a NaN. It's important to note that in Bayesian inference, there is no back-propagation step, and weight updates only occur when specific acceptance criteria are satisfied and therefore only the samples which do not have a NaN ever stand a chance of being accepted, and therefore the presence of NaNs brings down the efficiency. Both NUTS and HMC may suffer from this problem when we try to sample from an Isotropic Gaussian prior, but a Layer wise Scaled Gaussian Prior can helps to keep the variance of the weighted sums across the layers within stable bounds at each sampling step θ′. This is illustrated by the violin plot of the activations across the layers of the neural network used in the experiments (see Figures 3, 4). We observe that using a Layer wise Scaled Gaussian Prior reduces the variance of the activations and leads to Gaussian like unimodal distributed values across the layers for both the ReLU and the TanH activation functions. Thereby making our sampling process efficient, without the need to tune the variance.

In our study we have found the cold posterior effect to be absent in MCMC Bayesian neural networks on all the six binary classification dataset. On the two multi classification datasets where the training dataset is not balanced, we observe the cold posterior effect. The class weighted likelihood regains the better performance without cooling the posterior. This provides with an advantage in the cases where it may not be feasible to balance the datasets, such as the Yeast dataset, where out of 10 classes one class has only 5 samples, and we need some at-least 2 samples of it in the test data set. Using a class weighted likelihood and a Gaussian Prior does not suffer from the cold posterior effect and therefore, unlike Fortuin et al. (2021), we see no reason to discontinue using the Multivariate Gaussian priors if we assume that our likelihood i.e. the neural networks parameters follow a Normal distribution. In fact, using layer-wise scaled multivariate Normal Prior can facilitate a very efficient sampling process, making it accessible for a wide range of research.

## 5 Conclusion

The Gaussian Priors placed on the weights of a dense layer Bayesian Neural Network are found to be the most suitable choice among the four Priors, namely the Isotropic Cauchy, Isotropic Laplace, Isotropic Gaussian, and Layer wise Scaled Gaussian Priors. The heavy tailed Priors i.e. Isotropic Cauchy, and the Isotropic Laplace Prior suffer from inefficient convergence and are therefore not suitable. Within the choice of Gaussian Priors, the Layer wise Scaled Gaussian Prior is more efficient compared to an Isotropic Gaussian Prior.

The large variance of the Isotropic Gaussian Prior causes the activations to saturate and renders the inference process inefficient. *Layer wise Scaled Gaussian Priors* provides us with a consistent variance of the activations during the sampling process thus offering a speed up of upto four times and therefore we conclude that it should be the default choice when performing Bayesian inference of neural network parameters.

The Cold posterior effect was not observed in eight datasets, when using MCMC inference for neural networks, and the best classification performance is obtained at *T* = 1. We have found the class weighted likelihood to improve the classification performance and the convergence statistics, this is especially advantageous in datasets where balancing the datasets is not possible. Therefore we conclude that using layer-wise scaled Gaussian prior in conjunction with a class weighted likelihood can provide a default configuration for dense Bayesian neural networks, irrespective of the dataset. The improved sampling efficiency and classification performance comparable to state of the art make the MCMC Bayesian neural networks accessible for a wide range of research, especially for their advantage in cases where uncertainty quantification is required.

## Data Availability

The original contributions presented in the study are included in the article/Supplementary material, further inquiries can be directed to the corresponding author.

## Footnotes

^1^see Appendix A.1.5 for introduction to and definition of Glorot and He Initialization methods.

^2^Source code is provided as a zip file in the supplementary materials.

^3^for box plots showing the distributions of the weights across the network see Figures 2, 3 in the Appendix A.2
