# Supplementary material for: Layer wise Scaled Gaussian Priors for Markov Chain Monte Carlo Sampled deep Bayesian neural networks
Source: Front Artif Intell. 2025 Apr 25;8:1444891. doi: 10.3389/frai.2025.1444891 (PMC12061901; doi:10.3389/frai.2025.1444891)
Supplement: Supplementary file 2 [file Supplementary_file_1.pdf]

Appendix to the paper: Layer wise Scaled Gaussian Priors for Markov Chain Monte Carlo sampling (MCMC) Deep Bayesian Neural Networks

April 3, 2025

1 Appendix

1.1 Theory

1.1.1 Bayesian Neural Network

A Bayesian neural network can be defined as a neural network whose parameters $\theta = (W, b)$ are inferred using Bayesian Inference, and as a result are a distribution, namely the posterior. This distribution is in contrast to the point neural networks where the weights and biases are point estimates. In Figure 1 on the following page, we depict how such a distribution compares to the point neural network.

1.1.2 Bayesian Inference

Bayesian inference uses Bayes' theorem to update the hypothesis probability every time new data x is obtained. It calculates the posterior $P(\theta \mid x)$ based on a prior $P(\theta)$ and a likelihood probability $P(x \mid \theta)$ which is derived from a statistical model and observed data. In the context of computational sciences, the posterior can be understood as a distribution of weights and biases which best describe the given dataset. We can infer the posterior according to Bayes' theorem:

$$P(\theta \mid x) = \frac{P(x \mid \theta)P(\theta)}{P(x)} \tag{1}$$

where $\dots\theta$ stands for any hypothesis whose probability may be affected by data. Often there are competing hypotheses, and our task is to determine which is the most probable.

$P(\theta)$, the prior, is the probability of the hypothesis θ before the data x is observed.

$P(\theta \mid x)$ is the posterior which is the probability of θ after data x is observed. This is what we want to know

$P(x \mid \theta)$ is the likelihood is the probability of observing x for a fixed θ. It indicates the compatibility of the data with the given hypothesis. The likelihood function is a function of the data, x while the posterior is a function of the hypothesis θ.

$P(x)$ is called the evidence (probability of generating an observed sample from a prior) is sometimes termed the marginal likelihood or "model evidence". This factor is the same for all possible hypotheses being considered and hence does not factor into determining the relative probabilities of different hypotheses.

1.1.3 Markov Chain Monte Carlo sampling (MCMC)

Markov Chain Monte Carlo (MCMC) technique is the used for sampling the true posterior from complex probability distributions. MCMC algorithms construct a Markov chain that explores the posterior over multiple iterations. The chain(s) converge to the posterior, allowing us to generate samples from it. MCMC is versatile and can handle a wide range of probability distributions, including those that are not easily approximated by simpler distributions. However, MCMC techniques can be computationally intensive but they sample from the "true posterior" i.e. Equation (1).

Hamiltonian Monte Carlo (HMC) and No U-Turn Sampling (NUTS) leverage Hamiltonian dynamics to efficiently sample from the weight space. It involves calculating the following gradient.

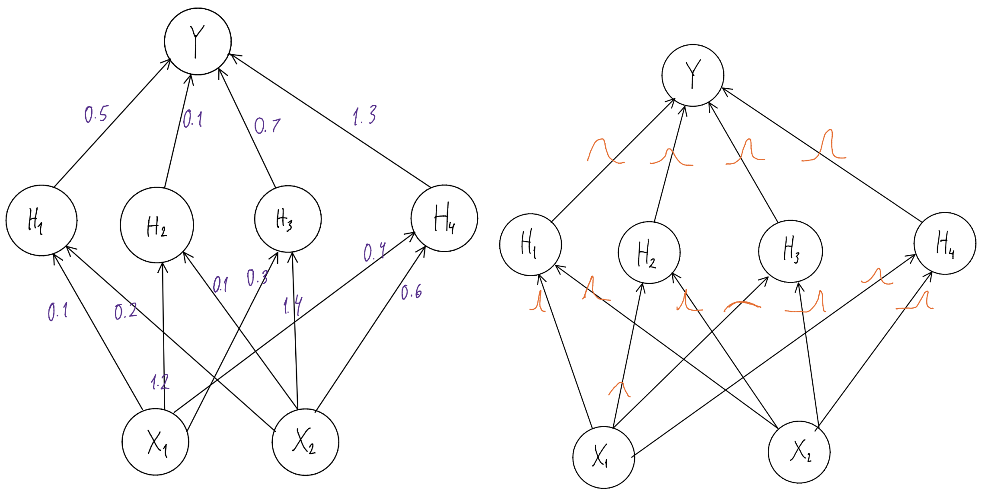

(a) Point (b) Bayesian

Figure 1: Unlike Point neural networks that have a fixed value of their parameters, Bayesian neural networks have a distribution defined over them

$$-\frac{\partial H}{\partial \theta} = \nabla_\theta(-\log P(x \mid \theta) - \log P(\theta)) \tag{2}$$

where, H stands for the Hamiltonian, θ is the set of weights and biases proposed at the current iteration, the $-\log P(x \mid \theta)$ is the negative log-likelihood term, i.e, at a given iteration step, how well does the θ explain the data. The log likelihood term can be written for a binary classification task of a dataset consisting of n points as follows [**?**],

$$\log P(x \mid \theta) = \sum_{i=1}^{n} (y_i \log \hat{y}_{\theta,i} + (1 - y_i) \log (1 - \hat{y}_{\theta,i})) \tag{3}$$

where, $\hat{y}_{\theta,i}$ is the prediction obtained for the ith sample by the neural network for a specific θ value and y_i is the corresponding true label of this sample.

During this training process we take a gradient of Equation (3) with respect to all the parameters θ. Taking the gradient of the loss function involves taking the gradient of the weighted sums contained in the expression $\hat{y}_{\theta,i}$. For binary classification problems the last layer of a network typically has a *sigmoid* activation function. If the weighted sums are very large or very small then they will result in the Sigmoid activation function saturating. We note that the implementation of the sigmoid function in most of the standard libraries clips the output to either 0 or 1 after a certain point and when this zero is inside the log, this quanity becomes undefined and this produces a NaN. It's important to note that in Bayesian inference, there is no back-propagation step, and weight updates only occur when specific acceptance criteria are satisfied and therefore only the samples which do not have a NaN ever stand a chance of being accepted, and therefore the presence of NaNs brings down the efficiency.

1.1.4 Neural Networks

In a deep learning process we try to fit the weights W and biases b of a neural network using the training data D, where D is composed of a series of input x and their corresponding labels y. Using a back-propagation algorithm, we can fit a set of parameters by minimizing a given cost function, one such cost (loss) function is the well known Negative Log-Likelihood given in Equation (3), where $\theta = \{W, b\}$ is the set of all the neural network parameters.

A gradient descent method such as the ADAM optimizer is used to achieve this optimization process. During this optimization process we take a gradient of Equation (3) with respect to all the parameters θ. Taking the gradient of the loss function involves taking the gradient of the weighted sums z contained in the expression $\hat{y}$. From a statistics point of view, we are performing what is called a Maximum Likelihood Estimation (MLE), or a Maximum A Posteriori (MAP) estimation when regularization is used.

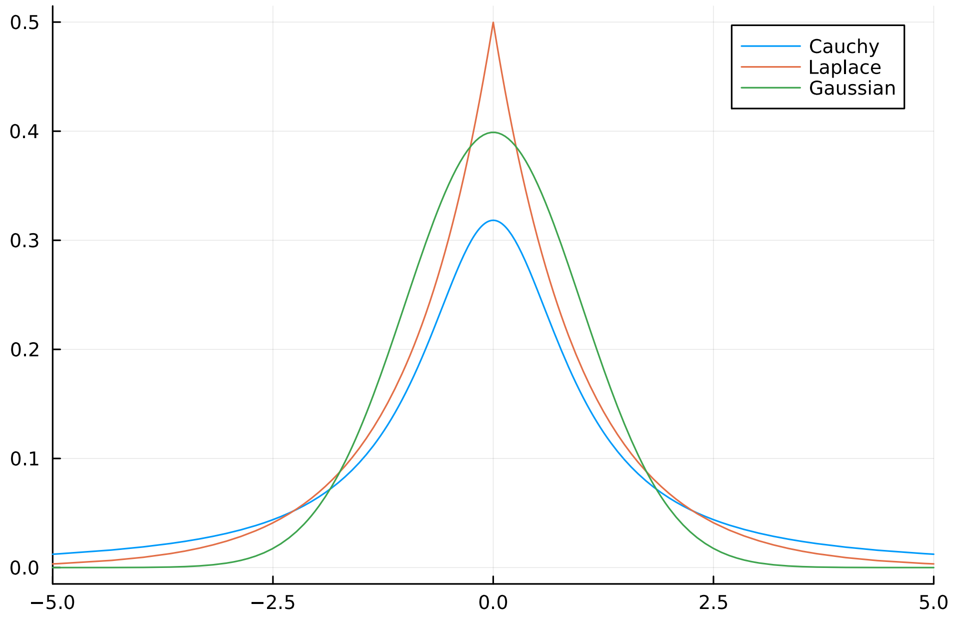

Figure 2: The three Isotropic Distributions, each with location 0 and scale 1

1.1.5 Initialization of Neural Networks

Given an untrained neural network, we have to start with a set of parameter values θ, where each of these typically ranging from zero to one. Naively, we could then randomly sample values from a Normal distribution, however we might suffer from the problem of vanishing, exploding, or unstable gradients. Vanishing gradients occur when the activations of the neurons become saturated and as a result the gradients calculated during the back-propagation step tend to zero and so the neural network is unable to learn. If the weights are very small, then the gradients will vanish and the weight updates will be so small that it would take an intractable amount of time for the training to progress. In the case of exploding gradients the weights of the network become very large, such that any weight change from one iteration to the next is very large, resulting in large gradients and large activations, thereby making the training unstable. Traditionally the most common strategy used to initialize the weights of a neural network is randomly sampling parameters from a normal distribution with a zero mean and unit variance. However this random initialization can cause unstable gradients because the variance of the output of a weighted sum is a function of three factors: the number of inputs to the weighted sum, the variance of the inputs, and the variance of the weights [?]. Consequently, if the relationship between the number of inputs to a weighted sum and the variance of the weights is incorrect, then the result of a weighted sum can have either a larger variance than the variance of its inputs or a smaller variance than the variance of its inputs, and if this difference across the layers is large enough then our training becomes unstable. Therefore, Glorot and Bengio [?] proposed initializing the weights of a sigmoid activated layer from a Normal distribution with a

$$\sigma^2 = 16\frac{2}{n_{in} + n_{out}} \tag{4}$$

where n_{in} is the number of input connections and n_{out} is the number of output connections for this layer. Similarly when using a ReLU activation, He-Initialization [?] maybe used, wherein we initialize the weights from a Normal distribution with a standard deviation of

$$\sigma^2 = 2\frac{2}{n_{in} + n_{out}} \tag{5}$$

These new changes introduced to the variance of the distribution which is used to initialize the layers of our networks, alleviate the problem of unstable gradients by keeping the variances of the weighted sums across the layers consistent.

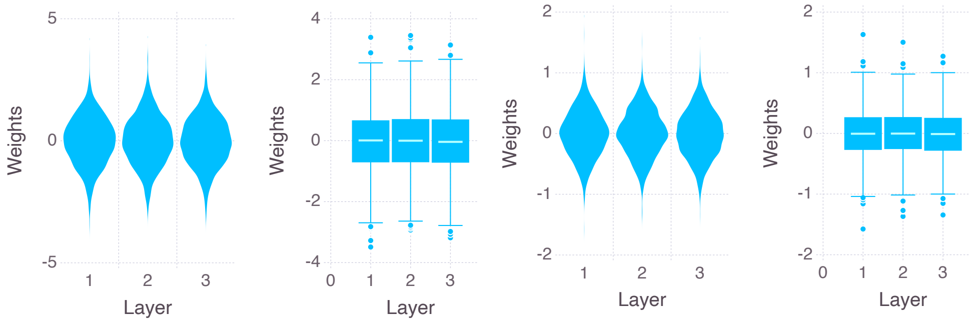

(a) Isotropic Gaussian prior (b) Layerwise scaled prior

Figure 3: Layer-wise distribution of ReLU activations after 2000 Monte Carlo Steps

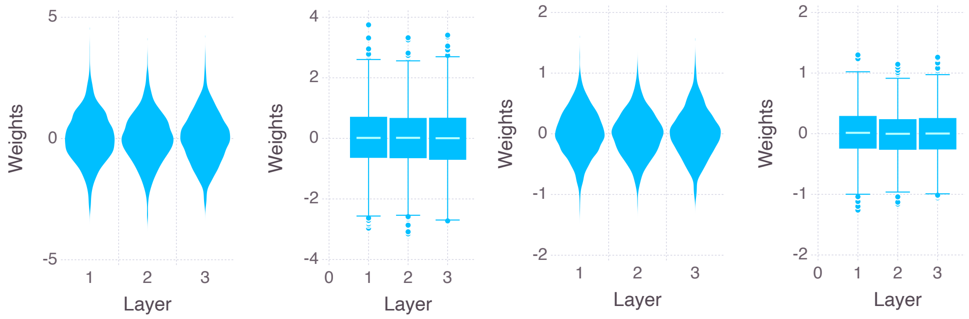

(a) Isotropic Gaussian prior (b) Layerwise scaled prior

Figure 4: Layer-wise distributions of TanH activations after 2000 Monte Carlo Steps

1.2 Additional Violin and Box plots

Here we show the violin plots and the box plots for the stroke dataset after the 200, 1000, and 2000 Monte Carlo steps of sampling. We obtain similar plots for the Iris and the Yeast datasets but they have been left out for brevity.

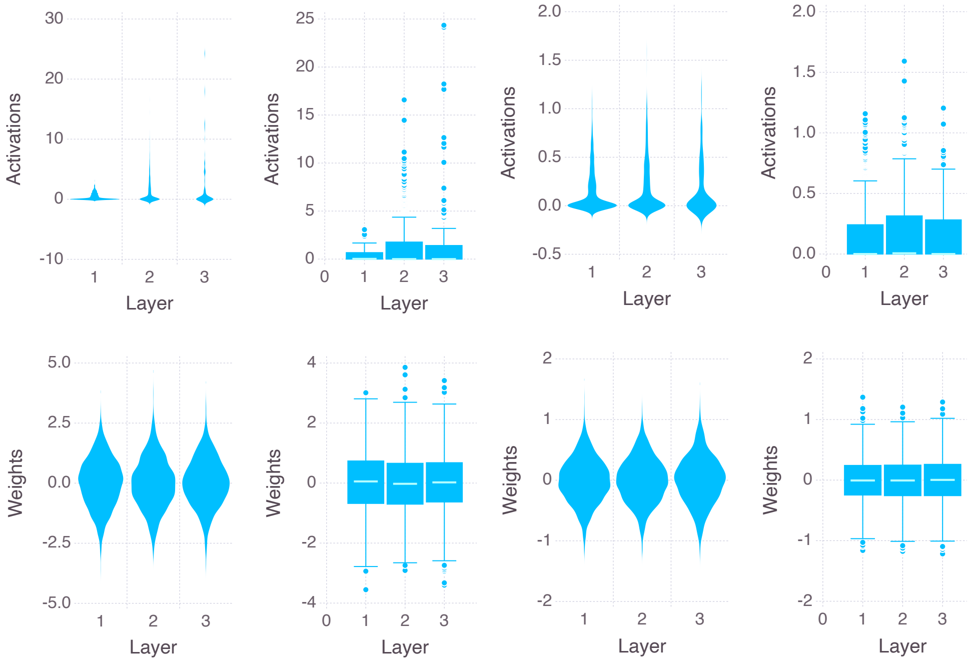

(a) Isotropic Gaussian prior (b) Layerwise Scaled Prior

Figure 5: Layer-wise distribution of ReLU activations after 200 Monte Carlo Steps

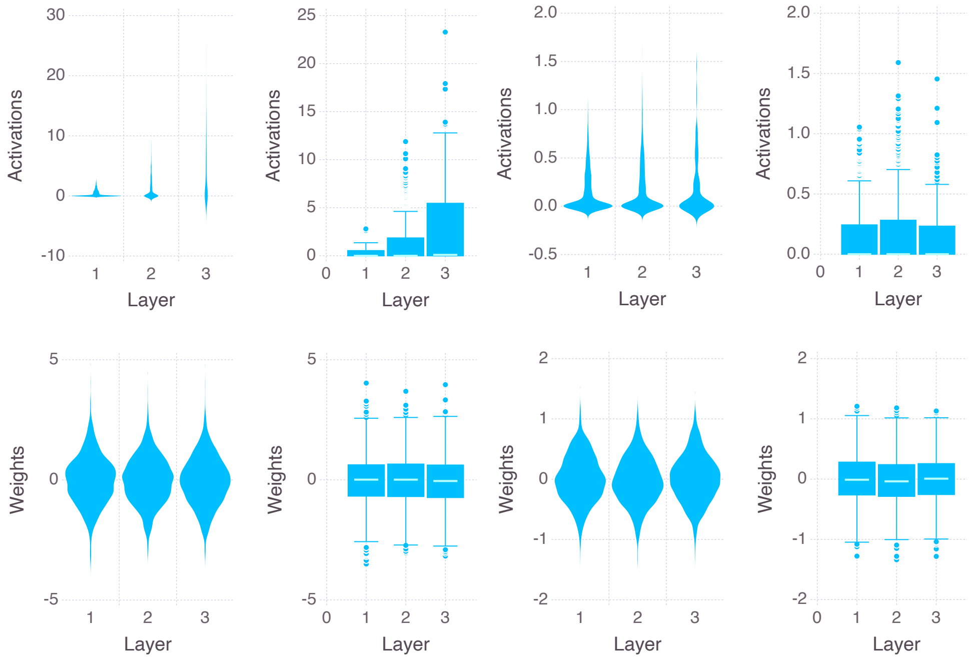

(a) Isotropic Gaussian prior (b) Layerwise scaled prior

Figure 6: Layer-wise distribution of ReLU activations after 1000 Monte Carlo Steps

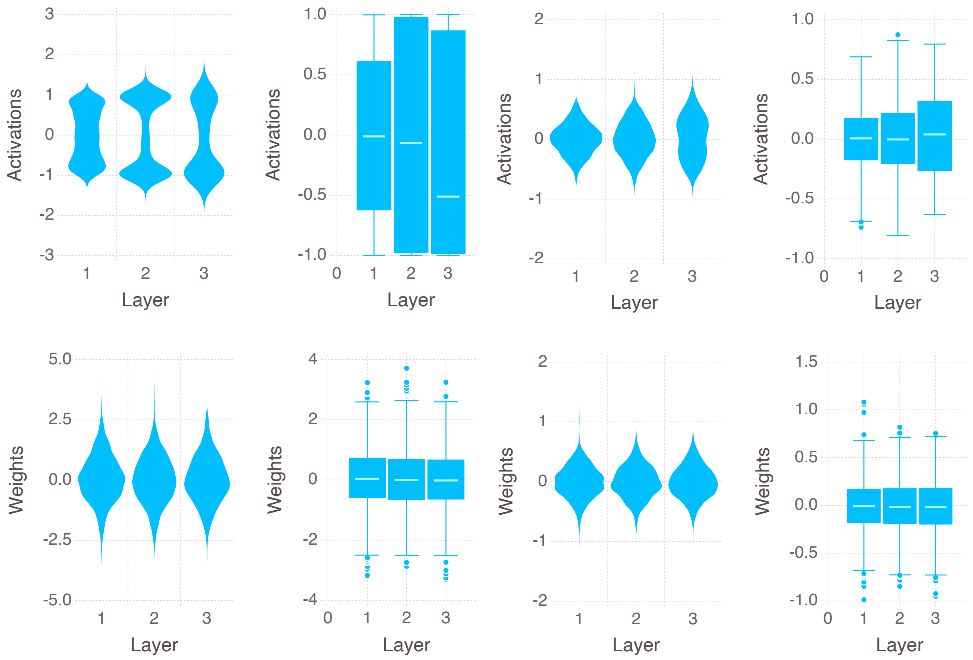

(a) Isotropic Gaussian prior (b) Layerwise Scaled Prior

Figure 7: Layer-wise distributions of TanH activations after 200 Monte Carlo Steps

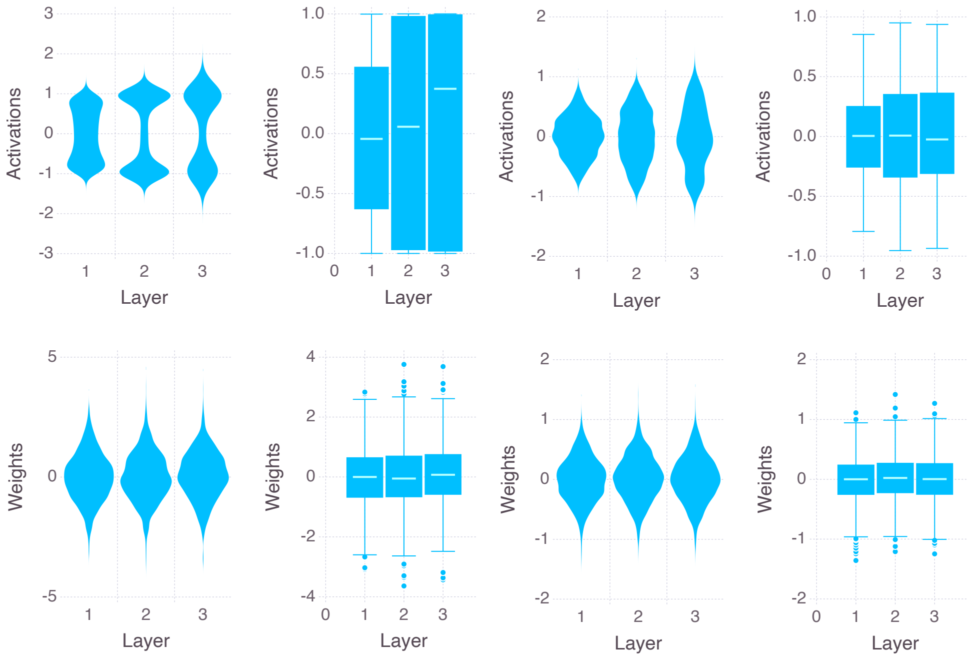

(a) Isotropic Gaussian prior (b) Layerwise scaled prior

Figure 8: Layer-wise distributions of TanH activations after 1000 Monte Carlo Steps
